# A renewed focus on primary health care: revitalize or reframe?

**DOI:** 10.1186/1744-8603-6-13

**Published:** 2010-07-30

**Authors:** Mrigesh Bhatia, Susan Rifkin

**Affiliations:** 1 Department of Social Policy, London School of Economics, Houghton Street, London, WC2A 2AE, UK; 2 Institute of Social Psychology, London School of Economics, Houghton Street, London, WC2A 2AE, UK

## Abstract

The year 2008 celebrated 30 years of Primary Health Care (PHC) policy emerging from the Alma Ata Declaration with publication of two key reports, the World Health Report 2008 and the Report of the Commission on the Social Determinants of Health. Both reports reaffirmed the relevance of PHC in terms of its vision and values in today's world. However, important challenges in terms of defining PHC, equity and empowerment need to be addressed.

This article takes the form of a commentary reviewing developments in the last 30 years and discusses the future of this policy. Three challenges are put forward for discussion (i) the challenge of moving away from a narrow technical bio-medical paradigm of health to a broader social determinants approach and the need to differentiate primary care from primary health care; (ii) The challenge of tackling the equity implications of the market oriented reforms and ensuring that the role of the State in the provision of welfare services is not further weakened; and (iii) the challenge of finding ways to develop local community commitments especially in terms of empowerment.

These challenges need to be addressed if PHC is to remain relevant in today's context. The paper concludes that it is not sufficient to revitalize PHC of the Alma Ata Declaration but it must be reframed in light of the above discussion.

## Introduction

In 1978, member States of the World Health Organization (WHO) attending the meeting in Alma Ata supported the policy of Primary Health Care (PHC) [1]. This policy shifted the focus of health improvements from mere provision of health services to the larger context of the relationship between health and social and economic development. Thirty years later, WHO and its regional affiliates called for PHC to be revitalised [2]. The purpose of this paper is to identify the major challenges to this call and investigate the commitments necessary to achieve this objective. It argues that today it is not sufficient to seek ways of revitalizing PHC. Rather it is necessary to reframe this policy to incorporate present issues arising from the national and global context.

## Background

PHC analyzed the reasons for health improvements beyond the technical biomedical intervention paradigm. It argued that other factors were equally important determinants. PHC in 1978 was underpinned by the concept of social justice and identified the main principles of equity and social justice as key to health improvements. It also highlighted the role of prevention, multisectoral collaboration, appropriate technology and sustainability. The need to improve the lot of those living in abject poverty was a major emphasis. PHC was a statement of values as much as a strategy for health care. The present call to "revitalize" PHC is to once again bring these values "to life; to animate" them [3].

It can be argued, however, that PHC in the global context of health care and health needs more than revitalization. It is necessary to re-"frame or shape" [3] PHC so that these principles can be translated from rhetoric into reality. The struggle to put policy to practice in PHC can be seen in the debate between Comprehensive and Selective PHC. The former argued that health improvements including those related to major diseases needed to be addressed in a context where health care delivery takes account of the principles and approaches described above. The latter argued to achieve PHC, it was necessary to focus on disease control targeting on diseases, which were more prevalent in terms of morbidity and mortality, and were cost effective [4].

The debate continues today. Comprehensive PHC has shown some remarkable successes, although it has not been a history of smooth progression. Notable examples of good programs have been seen in the NGO (non-government organizations) sector. These programs are often small scale projects run by charismatic leaders. Illustrations include Jamkhed in India which became a model for comprehensive PHC. It provided evidence of the value of Community Health Workers (CHWs) and a community development approach to health. Other examples can be found in the book by Taylor-Ide and Taylor [5].

On a national scale evidence is more restricted. The world's two most populated countries returned to PHC principles to address the health needs of the poor. China was the country that inspired PHC thinking through its attention to rural health care and the use of local people called "barefoot doctors" (CHWs) to give first line health care. After a period of market oriented reforms in health care and the resulting deterioration of the health of rural people who are the nation's population, China is now committing huge additional resources to revitalising rural networks based on PHC [6]. India, which was one of the first countries to create a national community health worker scheme after the Alma Ata conference and subsequently saw the scheme disappear within 10 years, has now begun to revive the program in the context of the National Rural Health Mission [7]. Thailand having adopted a "Basic Needs" approach in the 1970s established a health system based on an alliance between, government and NGOs that integrated PHC programs into other development programs. This alliance has produced both better health and economic improvements [8].

The year 2008 celebrated 30 years of PHC policy. Two major reports, the World Health Report 2008 [9] and the Report of the Commission on the Social Determinants of Health from WHO [10] provided key contributions to this celebration. Both reaffirmed the relevance of PHC in terms of its vision and values in today's world. In addition, a number of articles in the special issue of The Lancet [11] and in the Global Social Health Policy Forum written by public health researchers and activists summarise the influence of PHC on health policy [12]. However, at the risk of an understatement, the world has changed radically since 1978. The world in 2008, can be broadly described as one characterised by globalisation, rapid communication and an increasing gap between rich and poor. In the context of health and health care it can be described as one which has seen a shift from major concerns about communicable diseases to chronic diseases (from targeted single interventions to concerns about environment, life style and behaviour); ideological changes (as dictated by neoliberal economics and new public management) along with dominance of Bretton Woods institutions over the UN organisations resulted in developing countries embracing market oriented health sector reforms [13]; and a shift from medical professional monopoly on decisions and resource allocation to a much wider role for lay people [14]. This situation presents large challenges and demands serious rethinking about the PHC vision.

## Challenge One: Agreeing upon definitions

There is a challenge of getting a consensus among those involved in health care delivery and policy that health improvements must be seen in the context of linking health care and human development. In 1978, the idea of seeing health as a reflection of the wider socio-economic determinants was questioned by many of those working in the field of health. In putting forward the PHC policy, WHO used the personal experiences of people to give evidence of the link between health and development. Drawn mainly from less developed country experiences, those who contributed to this analysis were often charismatic doctors whose leadership and life improved the health of the poor with whom they worked. Coinciding with the widely reported achievements in health improvements in the newly created Peoples' Republic of China that viewed health as integral to development, the arguments that health was more than medicine and services gained credence among providers and policy makers. A book entitled "Health by the People" edited by Dr. Ken Newell, head of the division that crafted the PHC policy published proof of these accomplishments [15].

However, these views and arguments were not shared by the majority of those involved in health care. Many believed that there was little hard evidence to support the view that the socio-economic environment was as critical to health improvements as medicine and service delivery. As a result, although the arguments of social justice and equity were received with sympathy, implementation of policy mostly focused on service provision. One example is selective vs. comprehensive debate discussed above.

Another was the confusion of the concepts "Primary Health Care" and "primary care". Those concerned with health care delivery could embrace the PHC vision in the context of service delivery for which they were responsible. As a result their focus became providing first line health services, "primary care", for communities but not engaging in the wider analysis of conditions in which the poor health problems were created nor seriously engaging in activities to promote equity and community participation. The concept of primary care for many of these people was interchangeable with Primary Health Care and has continued to be so. A recent special issue of Lancet on 30 years of celebration of Alma Ata is a good example of the confusion in the understanding of differences between these two concepts [11]. A lack of a wider context for dialogue of the causes of and solutions to poor health creates considerable confusion for both policy planners and program implementers. In an attempt to clarify the relationship between Primary Health Care and primary care the Report of the Commission on the Social Determinants of Health states:

"The Alma Ata Declaration promoted Primary Health Care (PHC) as its central means toward good and fair global health--not simply health services at the primary care level (though that was important) but rather a health system model that acted also on the underlying social, economic and political causes of poor health". [[10]: pg.33].

A commitment to reject the duality between Comprehensive PHC and Selective PHC and an agreement for a standard definition of PHC and the attributes it encompasses is necessary to create solid frameworks for policy analysis and health promotion.

## Challenge Two: Ensuring equity

A second challenge is addressing the equity implications of the market oriented reforms introduced in number of developing countries. The Report of the Commission on the Social Determinants of Health highlighted equity, both in terms of distribution but also in terms of power and politics. Both PHC and the Report of the Commission call for universal coverage. They highlight problem of market oriented approaches and give evidence of its failure to meet objectives for improving health for the poor.

The reduced access to health care as a result of the Structural Adjustment Programs (SAP) of the 1980s provides the most graphic example illustrated by the reduction of life expectancy in Africa [16]. In the field of health, these programs combined with neo-liberal emphasis on the role of the market economy to improve efficiency and effectiveness has resulted in the promotion of a health system reforms (HSR). These market oriented reforms include decentralization, public private partnerships, promotion of the private sector, and introduction of user charges [17,18]. Although often couched in the PHC principles of equity and participation, they respond to the demands of efficiency at the cost of equity considerations [13]. As a result, short-term gains, in many cases, have overridden longer-term concerns that address the root cause of poverty and poor health [19].

Equity implications of the market oriented reforms are well documented. A classic example is the introduction of user charges. User charges for health care were introduced as a part of the structural adjustment programs in number of countries. However, the expected benefits in terms of efficiency and equity were not forthcoming. The negative consequences in terms of access and utilisation of health services were observed especially among the lower socio-economic groups across countries. Given the highly regressive nature of user charges and the lack of effective exemption mechanisms, it is therefore not surprising to observe number of countries in Sub-Sahara Africa have abolished user fees or are in the process of doing so [20].

At the global level, the global public private partnerships (i.e. Global alliance on vaccine initiative (GAVI), Global funds for Aids, TB and Malaria (GFATM) have funded technology for health focusing on profit rather than people and have re-enforced vertical disease program approach. This approach has been criticised for distorting national priorities, weakening the comprehensive integrated health systems approach and supporting re-verticalization of planning [21] as energies are directed towards implementing specific vertical disease programs.

## Challenge Three: Supporting community participation and empowerment

A final major challenge is to examine and seek ways forward to develop local community commitments. Community participation was identified as a key principle of PHC. There was little distinction between participation as community mobilisation (having community people accept professionals' assessment and activities for health improvements) and community empowerment (transforming attitudes and behaviours that enable community/individuals take decisions about their own lives). In recent years, recognition of the differences has increased and the term participation has increasingly been replaced by empowerment, calling attention to the importance of power and control over decisions, especially resource allocation.

The direct link between community participation and empowerment has not been easy to establish [22]. However the link has been strengthened by a recent systematic review undertaken by the Working Group on Community Based Primary Health of the American Public Health Association [23]. In a paper published in *Global Public Health *their findings show that community involvement including house to house visits by health staff, group meetings for education and support on health issues, outreach workers providing health services in the community and a community level (CHW) health worker to support community based health management has measurable effects on improvement of child survival. They also highlight empowering communities (meaning community people gain skills, information and confidence to make decisions about their choices affecting their own lives) as an overarching strategy that underpins these gains.

Issues around participation and empowerment also have been promoted in the context of governance of health service provision. A growing literature argues that concerns about accountability of public expenditures should be placed in the hands of those intended beneficiaries of those services. These issues centre on both the accountability of services to perform to the satisfaction of the users and the accountability of finances to be used in the way in which they have been allocated. Concerns are developed in discussion about "voice" whereby service users have the ability and capacity to demand the providers perform to user satisfaction. Evidence from the implementation of the Bamako Initiative shows how accountability can catalyze improvement in efficiency and effectiveness of local service delivery [24].

Commitments to meet this challenge continually demand professionals to hold serious dialogues with those for whom they provide service and care. To date this dialogue has often been delayed by several factors. Firstly, there is the existence of attitudes of professionals who tend to disregard opinions and views of those outside the profession. Secondly, there is the historic view that health interventions can only be verified by outcome measures. This view ignores the vital role of process in sustaining the improvements that bio-medicine and technology contribute. The World Health Report 2008 discusses in detail the role of service providers yet does not address the second issue of process. Commitment to addressing both issues is critical to PHC in the present context.

## Conclusion

Above we have identified the challenges and necessary commitments that need to be addressed if PHC is to remain relevant. Revitalizing PHC principles without developing a framework to address concrete measures for health improvements is not sufficient. The challenges discussed above need to be examined in a systematic and integrated way to produce flexible policy options and solutions that can be implemented. To do this, particularly in a time of financial crisis, requires a willingness to dialogue and appreciate a range of different and often contradictory views working toward consensus. It is clear that more of the same will not answer increasingly risky situations emerging from not only lack of money but also climate change, international political tensions and growing anxiety about resource availability due to rapidly expanding global populations. This does suggest that perhaps revitalizing PHC is not sufficient. What is needed is a reframing of the concept in light of the above discussions around the issues identified.

## Competing interests

The authors declare that they have no competing interests.

## Authors' contributions

Both authors have contributed to the planning, writing and editing of this paper. Both authors have read and approved the final version of this manuscript.

## References

[B1] Declaration of Alma-Ata International conference on primary health care Alma-Ata, USSR Sept 1978 6 12 http://who.int./hpr/NPH/doc/declaration_almaata.pdf (accessed 15 November 2009)

[B2] Pan American Health Organization Renewing Primary Health Care in the Americas 2007 Washington DC: PAHO 10.1590/s1020-49892007000200001 17565793

[B3] Oxford Shorter Dictionary 1973 3 Oxford: Oxford University Press

[B4] Rifkin S Walt G Why health improves: defining the issues concerning 'comprehensive primary health care' and 'selective primary health care' Soc Sci Med 1986 23 559 566 10.1016/0277-9536(86)90149-8 3764507

[B5] Taylor-Ide D Taylor C When communities own their future 2002 Baltimore: John Hopkins Univ Press

[B6] Yip W Hsiao W The Chinese Health System At A Crossroads Health Affairs 2008 27 2 460 468 10.1377/hlthaff.27.2.460 18332503

[B7] Government of India National Rural Health Mission document; Ministry of Health and Family Welfare New Delhi 2005

[B8] Chuengsatiansup K Srivanichakorn S Localizing global policy: the experience of Primary Health Care in Thailand Three decades of Primary Health Care: Reviewing the past and defining the future 2008 Bangkok: Prince Maidhol Award Conference http://www.pmaconference.org accessed August 9,2008

[B9] World Health Report Primary Health Care: now more than ever 2008 World Health Organization Geneva

[B10] Commission on the Social Determinants of Health Closing the gap in a generation 2008 World Health Organization Geneva

[B11] The Lancet Alma-Ata 30 years on: "Health for all need not be a dream buried in the past" The Lancet 2008 372 917 1007 10.1016/S0140-6736(08)61402-6

[B12] Koivusalo M Baru R Reclaiming Primary Health Care-Why does Alma Ata still matter? Or can we still speak of the relevance of Alma Ata? Global Social Policy Forum Global Social Policy 2008 8 2 147 166 10.1177/1468018108090611

[B13] Sen K Koivusalo M 'Health care reforms and developing countries - a critical overview' International Journal of Health Planning and Management 1998 13 199 215 10.1002/(SICI)1099-1751(1998070)13:3<199::AID-HPM518>3.0.CO;2-1 10187286

[B14] Florin D Dixon J "Public Involvement in Health Care" BMJ 2004 328 7432 159 161 10.1136/bmj.328.7432.159 14726350 PMC314519

[B15] Newell K Health by the people 1975 World Health Organization Geneva

[B16] Jolly R Adjustment with a human face: A UNICEF record and perspective on the 1980s World Development 1991 19 12 1807 1821 10.1016/0305-750X(91)90026-E

[B17] World Bank World Development Report: Investing in health 1993 Washington DC 8220523

[B18] Bhatia M Mossialos E Hall A, Midgley J 'Health Systems in developing countries'. In Social Policy for Development 2004 London: Sage 168 204

[B19] Green A Have health sector reforms strengthened PHC in developing countries? Primary Health Care Research and Development 2004 4 289 295 10.1191/1463423604pc219oa

[B20] Gilson L McIntyre D Removing user fees for primary care in Africa: the need for careful action British Medical Journal 2005 331 762 765 10.1136/bmj.331.7519.762 16195296 PMC1239983

[B21] Biesma R Brugha R Harmer A Walsh A Spicer N Walt G The effects of global health initiatives on country health systems: a review of the evidence from HIV/AIDS control Health Policy and Planning 2009 24 239 252 10.1093/heapol/czp025 19491291 PMC2699244

[B22] Hossain SM Bhuiya A Khan R Uhaa I Community development and its impact on health: South Asian experience BMJ 2004 328 830 833 10.1136/bmj.328.7443.830 15070644 PMC383386

[B23] Freeman P Perry HB Gupta SK Rassekh B Accelerating progress in achieving the millennium development goal for children through community-based approaches Global Public Health 2009 3 1 20 10.1080/17441690903330305 19890758

[B24] Mehrota S Jarrett S Improving basic health service delivery in low-income countries: 'voice' to the poor' Social Science and Medicine 2002 54 1685 1690 10.1016/S0277-9536(01)00336-7 12113450

